# Useful screening tools for preventing foot problems of diabetics in rural areas: a cross-sectional study

**DOI:** 10.1186/1471-2458-13-612

**Published:** 2013-06-27

**Authors:** Chia-Hao Chang, Yun-Shing Peng, Chang-Cheng Chang, Mei-Yen Chen

**Affiliations:** 1 Department of Nursing & the Chronic Diseases and Health Promotion Research Center, Chang Gung University of Science and Technology, Chiayi Campus, Chiayi, Taiwan; 2 Department of Respiratory Care, Chang Gung University of Science and Technology, Chiayi Campus, Taiwan; 3 Division of Endocrinology and Metabolism, Chang Gung Memorial Hospital, Chiayi, Taiwan; 4 Division of Plastic and Reconstructive Surgery, Chang Gung Memorial Hospital, Chiayi, Taiwan; 5 Graduate Institute of Nursing, Chang Gung University of Science and Technology, Chiayi Campus, Taiwan

**Keywords:** Diabetic foot problems, Michigan neuropathy screening instrument, Ankle brachial index, Receiver operating characteristic curves

## Abstract

**Background:**

Preventing diabetic foot problems (DFP) and their associated consequences is a critical in rural regions. The objective is to present an association of non-invasive DFP assessment tools and physiological indicators for early detection among rural cases of diabetes in Taiwan.

**Methods:**

Secondary data analysis of 387 participants previously diagnosed with type 2 diabetes was used. The Michigan Neuropathy Screening Instrument (MNSI), Ankle Brachial Index (ABI), optimal scaling combination (OSC) of MNSI, and age were used to examine peripheral neurovascular function. The King’s College classification (KC) and Texas risk classification (TRC) were used to understand diabetic foot complications.

**Results:**

The findings indicated that MNSI was negatively correlated with ABI, but positively with diabetes duration, age, KC, TRC, fasting blood glucose, low density of lipoprotein cholesterol, body mass index and waist circumference. The area under the receiver operating characteristic curves for assessing the risk of ABI based on OSC was larger than for MNSI, KC, and TRC.

**Conclusion:**

It is shown that using OSC, MNSI, and ABI as community screening tools is useful in detecting early neurovasculopathy. In addition, where an ABI machine is unavailable, primary healthcare providers that perform MNSI or OSC may be cost-effective. The study was approved by the institutional review board of the ethical committee (No 98-2224-B).

## Background

According to ICD-10, diabetes is the fifth most common cause of death, accounting for 35 deaths per 100,000 persons in 2008 [1]. The standardized mortality rate of diabetes in Taiwan is higher than that in the United States (14/100,000) and Japan (4/100,000). Diabetic foot ulcers occur in up to 25% of people with diabetes at some time in their life [2,3]. Diabetic foot problems (DFP) are a common cause of hospitalization worldwide, and are associated with lower limb amputation [1,4,5]. Sensory neuropathy is commonly associated with foot ulcers due to the loss of protective sensation. It is estimated that 45-60% of diabetic foot ulcers are purely neuropathic [6]. The mechanism of diabetic neuropathy may be associated with a decrease in myelinated fiber density, as hyperglycemia induces microvascular complications and loss or degeneration of nerve fibers. Peripheral arterial disease is a risk factor associated with diabetes foot complications, along with neuropathy, foot deformity, and infection [5,7]. DFP can be prevented via early screening and prompt treatment of peripheral neuropathy and vasculopathy [8]. Early symptoms are easily overlooked as this condition develops slowly [9]. Without adequate management, there is a high risk of infection and death among patients suffering from DFP. However, early prevention of DFP is often neglected in primary care settings in Taiwan, except in acute care settings.

Therefore, early screening for peripheral sensory neuropathy and peripheral circulation is important for diabetics, particularly among the rural elderly [9]. There are some efficient, inexpensive, and non-invasive measurement tools, such as monofilament and the vibration perception test that can assess peripheral sensory neuropathy [10,11]. MNSI is a reliable and valid scale for detecting diabetic neuropathy as reported [12]. However, it was not popularly used in primary health care settings. Furthermore, assessment of pedal pulses is a simple but valuable test to evaluate vasculopathy. Absent pedal pulses are evidence of peripheral vascular disease, even in asymptomatic patients [13,14]. Skin capillary circulation is more impaired in the toes of diabetic than non-diabetic patients with peripheral vascular disease [15]. Boyko et al. even suggested capillary refill time conveyed little diagnostic information and was omitted from routine foot examinations [16]. However, recently, study still stated that delayed capillary refill is indicative of ischemic disease. They referred increased capillary refill as a sign of venous insufficiency or obstruction [17]. Erasmus (2008) proposed that capillary refill time could be used to assess the perfusion of tissues in the feet [18]. In addition, KC has been used to focus on the development of foot ulcers [8,19]. It provides a management plan based on six stages of the condition. TRC uses risk factors that include the existence of ulceration, infection, Charcot foot, the degree of foot deformity and joint mobility, and the level of peripheral neuropathy and vascular together classify diabetic foot ulcer risks [20]. This provided a corresponding level of foot care according to eight categories. Previous studies have stated that TRC categories are highly associated with the increased risk of amputation and prolonged ulcer healing time [20,21]. In addition, ABI is used to assess blood flow and classify arterial disease [22,23]. The index is calculated by dividing the highest systolic pressure from the two pedal arteries by the brachial systolic pressure [4]. Values <0.9 were indicative of peripheral arterial disease [24]. Although ABI is a useful objective variable to assess DFP, an ABI machine is expensive (for example, the Cardio-Vision Model MS-2000 used in this study was ~$20,000).

There is good evidence that controlling blood glucose, pressure, and lipids can markedly reduce the adverse effects of diabetes and are substantially beneficial [25]. According to the recommendations for the target of diabetes treatment of the Taiwan Department of Health and authority organizations, the criteria for glycemic, lipid, and blood pressure measures [1,26] are fasting blood glucose <130 mg/dl, total cholesterol (TC) <175 mg/dl, and blood pressure <130/85 mmHg. As noted in previous study [27], they indicate that the prevalence of neuropathy increased with age. However, reports about the efficient community screening tools used for detection of the early stages of neurovasculopathy are limited. MNSI is a clinical scoring system developed as a quantitative instrument to document the presence and severity of diabetic peripheral neuropathy.

In accordance with the literature, we explore the relationship between MNSI, ABI, and health-related indicators among diabetes in the Western coastal region in Taiwan. Furthermore, we assessed whether the application of MNSI or a new index, based on the optimal scaling combination of MNSI and age (OSC), increases the area under ROC curves (AUCs) for assessing the risk of DFP in the elderly in Taiwan compared to KC and TRC. We hypothesized that MNSI or OSC is more significantly associated with the results of ABI evaluation than KC and TRC. Finally, we attempted to determine the cut-off points of MNSI and OSC for the diagnosis of DFP in the elderly.

## Methods

### Sample and setting

This study was part of a longitudinal cohort study of HPDMF (health promotion for preventing T2DM foot among rural community residents, 2009~2011) in the southwestern coastal Chia-Yi County. The original study adopted a multi-disciplinary team approach, including 12 certified nurses (three diabetes educators and nine public health nurses) and three senior doctors (an endocrinologist and plastic surgeons qualified for DM foot prevention and wound care). All research assistants and researchers (including the physicians and statistician) were given a three-day (eight-hour) training program including assessment procedures, familiarity with instruments, health promotion counseling techniques, and how to administer a structured interview. All participants received a three-stage professional health assessment over a one-year period. In this paper, we used secondary data analysis. Data were obtained from the first year study (2009). It included 387 Taiwanese community residents with T2DM, randomly selected from the local diabetes registration files by the public health nurses in each district. Simple random sampling from their local DM registration files was used by the PHNs in each of the nine districts. Selection criteria included subjects who: (1) were diagnosed as T2DM by a physician (2) had a stable physical condition with fasting glucose level less than 300 mg/dl and (3) were willing to participate in the study. Exclusion criteria were: (1) serious mental problems (2) serious complications of DM and (3) inability to walk to a local health center. On average, each district selected 40~50 participants.

### Instruments

(1) Peripheral neurological assessment was carried out using MNSI as previously described [18]. The diabetes nurse educators assessed five variables on both feet and counted the total points (MNSI, range 0~10): (1) Appearance of feet, if abnormal, then inspection of lower limbs for deformities, dry skin, fissure, calluses, or infection was carried out; (2) Identification of foot ulceration; (3) Vibration perception threshold testing - semi-quantitative assessment of vibration sensation was conducted with a 128-Hz turning fork on the dorsum of the big toe. Patients, whose eyes are closed, will be asked to indicate when they can no longer sense the vibration from the vibrating tuning fork. (4) Ankle reflexes were examined by tapping the Achilles tendon with a hammer; and (5) Touch-pressure sensation test with a 5.07/10 g Semmes-Weinstein monofilament by applying the monofilament perpendicular to the test sites of feet. The patients with closed eyes were asked to respond yes if they felt the filament. For this examination, it is important that the patient’s foot be supported (e.g. allow the sole of the foot to rest on a flat, warm surface). Abnormality was determined by the number of positive responses or abnormal clinical findings. Eight correct responses out of the 10 applications are considered as normal. A MNSI score greater than 2 (10-point scale) was considered neuropathic in this project and patients were referred to teaching hospitals for further evaluation [28]. According to Booth and Young [29], only 70% of the Semmes-Weinstein monofilaments buckled within +/-1.0 g of 10 g. Although Semmes-Weinstein monofilaments were not a perfect instrument, it was popularly used in Taiwan. Further research is necessary to validate the test. The MNSI procedure took 6–8 minutes for each participant.

(2) Peripheral vascular assessment: three variables were used to assess peripheral vascular function by trained nurses. (a) The Cardio-Vision Model MS-2000 was used to detect ABI, assessed by research nurses. ABI was calculated from ankle pressure/arm pressure. A low ankle-arm index is a good marker of vascular disease. Values of ABI were classified as ≥0.9 normal and <0.9 abnormal [24]. The ABI test is a popular tool for the non-invasive assessment of peripheral arterial disease with many supported data in the past. It is known to be unreliable on patients with arterial calcification and results in less or incompressible arteries, which is often found in patients with diabetes. Nevertheless, the ABI is a fast and painless exam with quantitative ratio presentation rather than manual measurement. (b) Palpable pedal, posterior tibias, and popliteal pulses were recorded as absent, weak or present. (c) Capillary refill time was done by pressing the tip of the toenail for two seconds, and taking the time for the blanched area to turn pink again. If the return time took >2 seconds, this was taken as ischemia. Assessing of all three variables of peripheral vascular assessment took 10–15 minutes for each participant.

(3) Diabetic foot risk assessment was assessed by plastic surgeons: (a) KC contained six stages of condition: not at risk, at risk, ulcer, cellulites, necrosis, and amputation [19]. (b) The TRC system was divided into six categories in origin [20]. We re-categorized three levels: low risk, at risk, and high risk because in a community settings, most of the participants were categorized in the first two levels.

(4) The blood glucose, TC, and low density of lipoprotein cholesterol (LDL) were drawn from the last 1–2 month diabetes passport record for each participant. Blood pressure was measured according to standard procedures by nurses during the study. BMI was calculated for each participant using the standard formula (weight in kilograms divided by square of the height in meters). Waist circumference (WC) in centimeters were used to measure central obesity, measuring the mid-abdominal distance between the last rib margin and the iliac crest.

(5) OSC, the optimal scaling combination of MNSI and age, which was derived from a logistic regression model.

### Procedure and ethical considerations

The study was approved by the institutional review board of the ethical committee (No 98-2224-B). Informed consent was obtained from all participants. Participants were notified about the survey by public health nurses and had the opportunity to review the questionnaire and indicate if they did not want to participate in this study. For ethical reasons, a cover letter was sent with the questionnaire, emphasizing that the responses are confidential. During the data analysis, confidentiality was maintained by data coding.

### Analysis

SPSS (Version 14.0) was used for data analyses. All tests were 2-sided and p-values <0.05 were considered statistically significant. Categorical data analyses (chi-square test, odds ratios and 95% confidence intervals) were applied to identify the association of MNSI, ABI and health-related indicators. Of the 391 participants selected, 387 completed the study. Missing data were excluded from analysis. Four of the missing data included 2 participants were type1 diabetes and 2 participants uncompleted biomarkers were examined. OSC was derived from a logistic regression model. The formula of OSC was taken as Eq. (1)

(1) OSC=0.1×age+0.2×MNSI

We computed the AUCs for the identification of the results of ABI evaluation by using KC, TRC, MNSI, and OSC to evaluate the index that had the highest association with the results of ABI evaluation. The area test has been applied to test the equality of ROC curves [30]. Based on the significance of the p-values from the area test, we identified the best predictor of the results of ABI evaluation in terms of sensitivity and specificity. An MNSI score of 2, KC of 2, and TRC of 2 (based on a previous study) were taken as cut-off values [19,20,28]. The optimal cut-off value of OSC, MNSI, KC, and TRC was calculated as Eq. (2)

(2) $$\begin{array}{l} \text{Optimal}\;\text{cut}-\text{off}\;\text{value}\\ \;=\text{min}\left(\sqrt{{\left(1-\text{sensitivity}\right)}^{2}+{\left(1-\text{specificity}\right)}^{2}}\right) \end{array}$$

## Results

### Demographic and the relationship between MNSI, ABI, and health-related indicators

**Figure 1:**
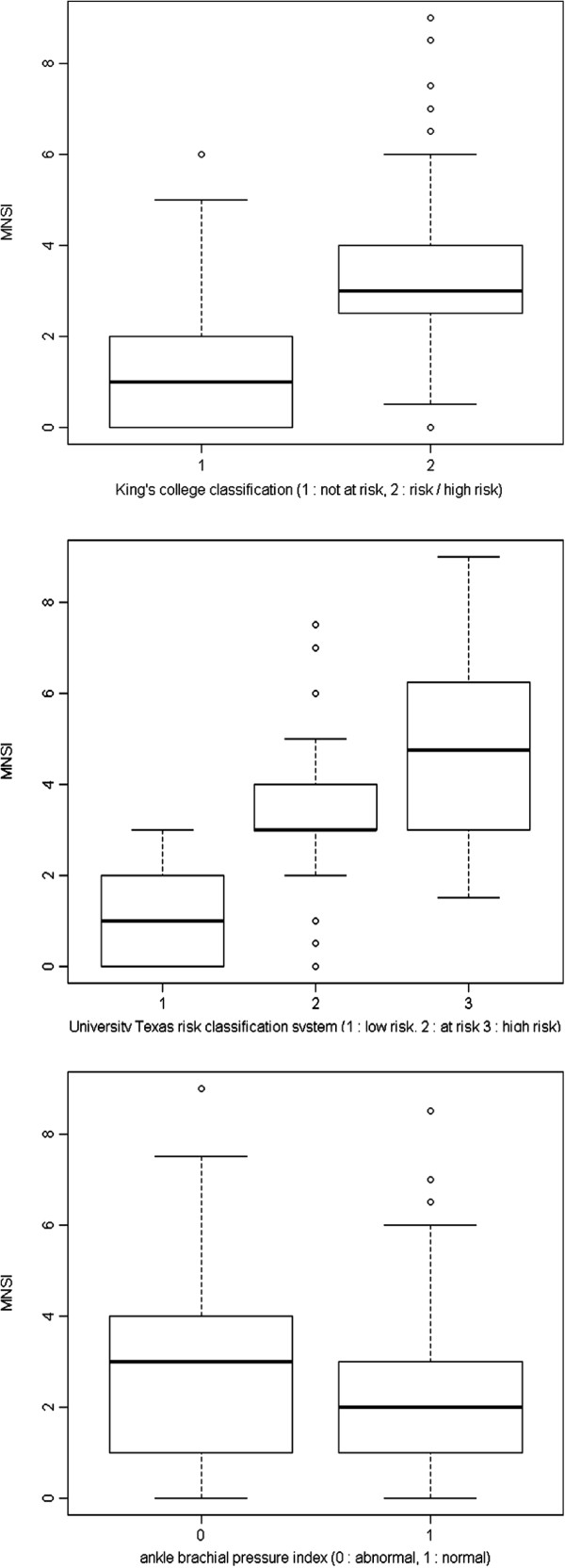
Box plots of MNSI versus ABI, KC and TRC.

A total of 59% of participants were female, the mean age of the participants was 68.7 (±9.4) years. The average of diabetes duration since diagnosis was 8.17 (± 6.30) years (Table 1). Regarding to foot examination, 43.2 % of the participants (n = 140) had a MNSI > 2.0. In addition, an ABI < 0.9 was noted in 10.8% participants (n = 42). Tables 2 and 3 show the MNSI score was significantly negatively associated with both sides of ABI. In addition, MNSI was significantly positively associated with diabetes duration, age, KC, TRC, blood glucose, LDL, BMI, and WC. Figure 1 presents the plots of the relationship.

**Table 1:** Demographic characteristics (N=387)

**Variables**	**Mean (SD, Range)**	**N %**
Age (year)	68.7 (9.4, 27~ 90)	
Duration of diabetes year	7.9 (6.2, 0.5~33)	
Participated DM support group		
Yes		195 (50.4)
No		192 (49.6)
Adopted oral medications		
Yes		351 (90.7)
No		36 ( 9.3)
Adopted insulin injection		
Yes		20 ( 5.2)
No		367 (94.8)
Fasting blood glucose		
≦130		135 (34.9)
≧131		226 (58.4)
missing		26 ( 6.7)
HbA1C		
≦6.99%		26 ( 6.7)
≧7.00%		221 (57.1)
missing		75 (19.4)
BMI		
Normal (~24)		118 (30.5)
Overweight (~27)		123 (31.8)
Obesity (>27)		146 (37.7)
Waist circumference (cm)		
Normal (male≦90, female≦80)		129 (33.3)
Abnormal (male≧91, female≧81)		258 (66.7)
Michigan Neuropathy Screening score		
≦3.0		301 (77.8)
≧3.5		86 (22.2)
ABPI (ankle brachial pressure index)		
≧ 0.90		338 (87.3)
≦ 0.89		49 (12.7)
Systolic Blood pressure		
≦130		121 (31.3)
≧131		266 (68.7)
Diastolic Blood pressure		
≦85		299 (77.3)
≧86		88 (22.7)
Exercise behavior		
Regular		228 (58.9)
Irregular		159 (41.1)
Adopted diabetes diet		
Yes		256 (66.1)
No		131 (33.9)
Oral care (Brush after meal )		
Yes		129 (33.3)
No		258 (66.7)
Oral hygiene-using dental floss		
Yes		135 (34.9)
No		252 (65.1)
Number of teeth		
~10		249 (64.3)
~20		60 (15.5)
~28		78 (20.2)
Depression score		
≦12		320 (82.7)
≧13		67 (17.3)

**Table 2:** The relationship between MNSI, ABI and health-related physiological indicators

	**MNSI**^**a**^	**ABI**	
		**Right side**	**Left side**
ABI^b^			
Right side ABI	-.19***		
Left side ABI	-.17***	.80***	
DM duration (years)	.12*	- .10*	- .14**
Age	.29***	-.23***	-.19***
King’s college classification	.58***	-.29***	-.26***
Texas risk classification^c^	.72***	-.25***	-.23***
Systolic blood pressure	.07	-.17**	-.15**
Diastolic blood pressure	.01	.01	.03
Blood glucose	.14**	-.03	-.02
TC	.10	-.07	-.09
LDL	.25***	- .05	-.12*
Body mass index	.11*	.10	.09
Waist circumference	.22***	.03	-.03

^a^ MNSI Michigan neuropathy screening instrument ^**b**^ ABI Ankle brachial index ^c^ Texas risk classification: University of Texas diabetic foot risk classification *p < .05, **p <.01, ***p < .001.

**Table 3:** The relationship between MNSI and health-related physiological indicators

**Variables**		**MNSI**^**a**^		**χ**^**2**^	**Odds ratio**	**95%**
	**Normal**		**Abnormal**			
		**N (%)**		**(d.f.=1)**		**Confidence interval**
Gender						
Female	141 (62.1)		86 (37.9)	6.49**	1.70	1.13~2.57
Male	78 (49.1)		81 (50.9)			
Education						
<= primary school	162 (52.1)		149 (47.9)	14.07***	0.34	0.19~0.61
>= secondary school	57 (76.0)		18 (24.0)			
ABI^b^						
Normal	198 (58.9)		138 (41.1)	5.10*	2.01	1.09~3.71
Abnormal	20 (41.7)		28 (58.3)			
King’s college classification^c^						
Normal	179 (82.1)		39 (17.9)	129.90***	14.33	8.75~23.47
Risk and high risk	41 (24.3)		128 (75.7)			
Texas risk classification^d^						
Low risk	182 (96.3)		7 ( 3.7)	234.35***	109.47	47.56~251.98
At/ or high risk	38 (19.2)		160 (80.8)			
Right side dorsal pedal pulses^e^						
Present/weakness	180 (60.2)		119 (39.8)	6.49*	1.86	1.15~3.01
Absent	39 (44.8)		48 (55.2)			
Left side dorsal pedals pulses						
Present/weakness	171 (60.9)		110 (39.1)	7.14**	1.85	1.17~2.90
Absent	48 (45.7)		57 (54.3)			
Right side poster tibias pulses						
Present/weakness	179 (59.1)		124 (40.9)	3.14	1.55	0.95~2.53
Absent	40 (48.2)		43 (51.8)			
Left side poster tibias pulses						
Present/weakness	174 (59.0)		121 (41.0)	2.58	1.47	0.92~2.36
Absent	45 (49.5)		46 (50.5)			
Right side popliteal pulses						
Present/weakness	163 (59.3)		112 (40.7)	2.51	1.43	0.92~2.23
Absent	56 (50.5)		55 (49.5)			
Left side popliteal pulses						
Present/ weakness	163 (59.1)		113 (40.9)	2.13	1.39	0.89~2.17
Absent	56 (50.9)		54 (49.1)			
Right capillary refill time						
<2 seconds	191 (59.5)		130 (40.5)	3.79	1.76	0.99~3.14
>2 seconds	25 (45.5)		30 (54.5)			
Left capillary refill time						
<2 seconds	190 (59.0)		132 (41.0)	2.61	1.61	0.90~2.89
>2 seconds	25 (47.2)		28 (52.8)			

^a^ MNSI Michigan Neuropathy Screening Instrument: normal <2 ^**b**^ ABI any side of ankle brachial index <0.9 was defined as abnormal. ^**c**^ King’s college classification: in this table, except the level of not at risk, we re-categorized the other five conditions of at risk, ulcer, cellulites, necrosis and amputation as risk and high risk. ^d^ University Texas risk classification system: in this table, we re-categorized the six conditions of at risk, ulcer, cellulites, necrosis and amputation as risk and high risk. ^e^ Palpable pulses between both sides of dorsal pedals, poster tibias, and popliteal areas. *p < .05, ** *p*<.01, ***p < .001.

### AUCs for the identification of the results of ABI with MNSI, OSC, KC, and TRC

**Figure 2:**
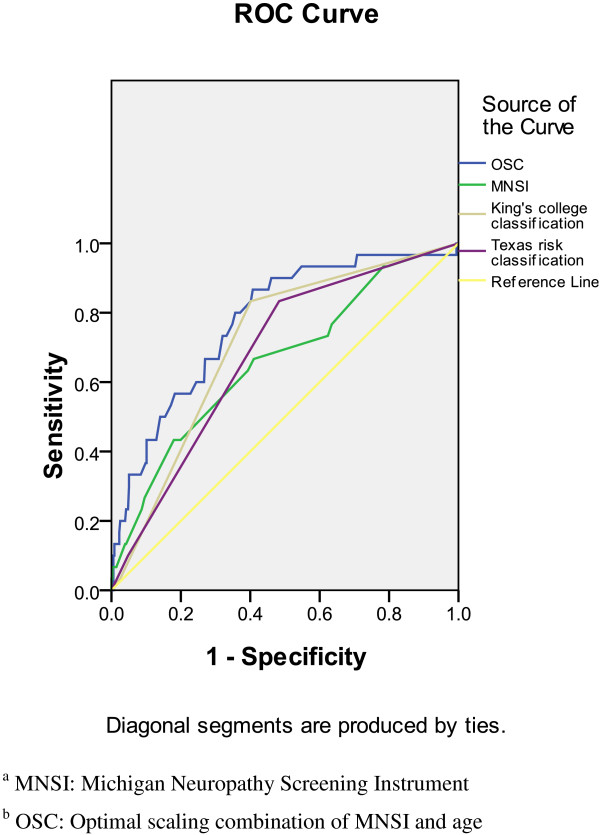
The ROC curves for the identification of ABI with KC, TRC, and MNSI.

Table 4 shows the results of the area test. The area under the ROC curve based on OSC was significantly larger than the area based on MNSI (OSC vs. MNSI, p < 0.05). However, the AUC based on MNSI was not significantly different with the area based on KC (KC vs. MNSI, p = 0.361) or TRC (TRC vs. MNSI, p = 0.69). These results imply that KC and TRC were not significantly related to the results of ABI evaluation than MNSI. However, OSC was more significantly related to the results of ABI evaluation than MNSI. Figure 2 shows the ROC curves for the identification of the results of ABI evaluation with OSC, MNSI, KC, and TRC. Apparently, the AUC for OSC was higher than that for MNSI, KC, and TRC. Table 4 shows the sensitivity and specificity for the identification of the results of ABI evaluation with cut-off values for MNSI, OSC, KC, and TRC. The variables are arranged as follows from high to low sensitivity: KC >1, TRC >1, OSC ≥7.6, and MNSI ≥2. The variables are arranged as follows from high to low specificity: OSC ≥7.6, KC >1, TRC >1, and MNSI ≥2.

**Table 4:** **AUCs, area test, sensitivity, and specificity for the prediction of the abnormal ABI**^**a**^

	**AUC (95% C.I.)**	**p-value for area test**	**Sensitivity**	**Specificity**
MNSI^b^	0.660 (0.554, 0.765)	-	0.8	0.6433
OSC^c^	0.775 (0.69, 0.86)	0.0154	0.7333	0.3764
KC^d^	0.712 (0.626, 0.798)	0.361	0.8333	0.5984
TRC^e^	0.679 (0.589, 0.770)	0.69	0.8333	0.5169

^a^ ABI any side of ankle brachial index <0.9 was defined as abnormal. ^b^ MNSI Michigan Neuropathy Screening Instrument: normal <2 ^c^ OSC Optimal scaling combination: normal <7.6 ^d^ KC: King’s college classification: normal <2 ^e^ TRC University Texas risk classification system: normal <2.

## Discussion

Peripheral vascular disease is a frequent long-term complication and the principal cause for treatment of hospitalized diabetic participants [31]. More specifically, abnormal pressure distribution and sensory deficit are the primary causes for diabetic patients developing foot problems. Vascular damage with decreased oxygen supply to the peripheral nerves can lead to the death of nerve tissues, adding to the development of these lesions. By taking nerve biopsies, a correlation between the presence and the degree of microvascular abnormalities with the presence and severity of diabetic neuropathy has already been reported [32,33].

### MNSI correlation with ABI, KC, TRC and diabetic related health variables

We have shown a strong relationship between MNSI and ABI. KC and TRC are clinically significant diabetic foot ulcer classification systems. The significant relationship between MNSI and KC, and MNSI and TRC, demonstrate that MNSI is a useful screening tool for preventing DFP. Systolic blood pressure is a known indicator associated with vascular damage in diabetic disease. Meanwhile, diabetic-related health variables, such as blood glucose, LDL, and WC, are positively associated with the severity of diabetic neuropathy. These relationships have been demonstrated with Pearson or Spearman correlation coefficients in a similar way in this study.

### ROC for the prediction of the results of ABI is higher if OSC is used

Aging is a known indicator in the development of DFP [34]. This association was evident in our study to show the importance of both in vascular and nerve function. MNSI is the most cost-effective community screening tool for peripheral neurological assessment and ABI is the first-line study assessing peripheral vascular function. However, age is an important indicator that should be considered before using MNSI or ABI as an indicator, because aging can increase the risk of developing DFP. Combining data on age and MNSI more closely correlates with the ABI for rural community diabetes. In fact, the use of only ABI or MNSI for all individuals may lead to an underestimation of DFP in the elderly.

To diagnosis DFP, we propose the use of OSC as a useful screening indicator for identifying an optimal cut-off point. Table 4 results show that the AUC for the prediction of the results of ABI among rural community diabetes is higher when OSC is used. Since finding a simple screening method for DFP was the major purpose of this study, it was important to determine which indicator was the most effective for ABI assessment. Figure 2 and Table 4 show ROC curves and sensitivity as well as specificity for the identification of the results of ABI evaluation, with cut-off values for MNSI, OSC, KC, and TRC. Note that, the optimal cut-off values (MNSI ≥2, and KC, TRC >1) are the same as the original criteria from previous references. The OSC showed a higher ROC curve and a balance of sensitivity and specificity for the prediction of the abnormal ABI, which are 80.0% and 64.3%, respectively. This result implies that age is a core criteria for DPF in rural community diabetes.

## Conclusions

We have investigated the relationships between the noninvasive tools of MNSI, ABI, and diabetic-related health variables, and to prevent early diabetic foot problems among rural community diabetic patients. This study has four principal findings: (1) MNSI is correlated with vascular function and diabetic-related health variables; (2) ABI is associated with peripheral nerve function and health variables; (3) this is the first study to combine age with MNSI as a new index (OSC) to evaluate peripheral nerve function; and (4) using MNSI and OSC as community screening tools, because they are cost-effective and useful in preventing DFP.

MNSI, OSC, KC, and TRC are useful screening tools for revealing the incidence of peripheral neurovasculopathy or DFP among the elderly. ABI is the most useful objective variable in assessing problems with arterial blockage and blood flow. Based on the AUCs for the identification of the results of ABI evaluation, we suggest that OSC is a better predictor of DFP in the elderly. OSC cut-off points can be converted into a consumer-friendly table (Additional file 1). The results of this study suggest that primary healthcare providers should apply OSC to screen and improve poor health and lifestyles in elderly diabetics in rural communities. Since we knew the argument of false negative in accuracy, we studied the correlation with the MNSI and OSC of MNSI to evaluate the ABI role on screening in rural population. Probably for the next step, we will apply other tools to obtain the quantitative data, such as skin perfusion pressure. Further research should consider other ethnicities and self-management intervention for elderly diabetics in rural communities since this could be a valuable procedure for improving their health.

## Competing interests

The authors declare that they have no competing interests.

## Authors’ contributions

MYC designed the study, and prepared the manuscript. CHC did the statistical analysis, and drafting of the article. YSP and CCC participated in and carried out the field work. All authors read and approved the final manuscript.

## Pre-publication history

The pre-publication history for this paper can be accessed here:

http://www.biomedcentral.com/1471-2458/13/612/prepub

## Supplementary Material

Additional file 1 Reference table for OSC. Click here for file
