# Supplementary material for: Useful screening tools for preventing foot problems of diabetics in rural areas: a cross-sectional study
Source: BMC Public Health. 2013 Jun 27;13:612. doi: 10.1186/1471-2458-13-612 (PMC3848866; doi:10.1186/1471-2458-13-612)
Supplement: Additional file 1 — Reference table for OSC. [file 1471-2458-13-612-S1.pdf]

Reference table for OSC

age / MNSI	0.0	0.5	1.0	1.5	2.0	2.5	3.0	3.5	4.0	4.5	5.0	5.5	6.0
65	6.5	6.6	6.7	6.8	6.9	7.0	7.1	7.2	7.3	7.4	7.5	7.6	7.7
66	6.6	6.7	6.8	6.9	7.0	7.1	7.2	7.3	7.4	7.5	7.6	7.7	7.8
67	6.7	6.8	6.9	7.0	7.1	7.2	7.3	7.4	7.5	7.6	7.7	7.8	7.9
68	6.8	6.9	7.0	7.1	7.2	7.3	7.4	7.5	7.6	7.7	7.8	7.9	8.0
69	6.9	7.0	7.1	7.2	7.3	7.4	7.5	7.6	7.7	7.8	7.9	8.0	8.1
70	7.0	7.1	7.2	7.3	7.4	7.5	7.6	7.7	7.8	7.9	8.0	8.1	8.2
71	7.1	7.2	7.3	7.4	7.5	7.6	7.7	7.8	7.9	8.0	8.1	8.2	8.3
72	7.2	7.3	7.4	7.5	7.6	7.7	7.8	7.9	8.0	8.1	8.2	8.3	8.4
73	7.3	7.4	7.5	7.6	7.7	7.8	7.9	8.0	8.1	8.2	8.3	8.4	8.5
74	7.4	7.5	7.6	7.7	7.8	7.9	8.0	8.1	8.2	8.3	8.4	8.5	8.6
75	7.5	7.6	7.7	7.8	7.9	8.0	8.1	8.2	8.3	8.4	8.5	8.6	8.7
76	7.6	7.7	7.8	7.9	8.0	8.1	8.2	8.3	8.4	8.5	8.6	8.7	8.8
77	7.7	7.8	7.9	8.0	8.1	8.2	8.3	8.4	8.5	8.6	8.7	8.8	8.9
78	7.8	7.9	8.0	8.1	8.2	8.3	8.4	8.5	8.6	8.7	8.8	8.9	9.0
79	7.9	8.0	8.1	8.2	8.3	8.4	8.5	8.6	8.7	8.8	8.9	9.0	9.1
80	8.0	8.1	8.2	8.3	8.4	8.5	8.6	8.7	8.8	8.9	9.0	9.1	9.2

1. The blue area represents low risk.
2. The pink area represents DFP risk.
3. OSC: Optimal scaling combination: normal <7.6
